# Health-promoting lifestyle as a predictor of well-being in Honduran university students: a structural equation modeling approach with mental health and sleep quality as mediators

**DOI:** 10.3389/fpsyg.2025.1735602

**Published:** 2026-01-20

**Authors:** Marcio Alexander Castillo-Díaz, Carlos Alberto Henao Periañez

**Affiliations:** 1 Graduate Program in Psychometrics and Educational Evaluation, Department of Psychology, Faculty of Social Sciences, Universidad Nacional Autónoma de Honduras, Tegucigalpa, Honduras; 2 School of Nursing, Faculty of Health, Universidad del Valle, Cali, Colombia

**Keywords:** anxiety, depression, health behavior, sleep quality, structural equation model, university students, well-being

## Abstract

**Introduction:**

Although a health-promoting lifestyle is associated with greater well-being among university students, the psychological (anxiety, depression) and physiological (sleep quality) mechanisms underlying this relationship remain insufficiently established, highlighting the need for integrative models that better explain well-being. Moreover, empirical evidence on these relationships is still limited in lower–middle-income countries. Accordingly, this study tested a model in which a health-promoting lifestyle predicts subjective well-being, with depression, anxiety, and sleep quality serving as mediating variables within a Latin American context.

**Method:**

A cross-sectional design was employed with a sample of 6,704 Honduran university students. The instruments included a short version of the Health-Promoting Lifestyle Profile II (HPLP-II), the Patient Health Questionnaire-9 (PHQ-9), the Generalized Anxiety Disorder 7-item Scale (GAD-7), the Single-Item Sleep Quality Scale (SQS), and the World Health Organization Well-Being Index (WHO-5). Measurement models for each instrument and a multiple-mediation structural equation model were estimated.

**Results:**

The final structural model demonstrated adequate fit and explained 61.6% of the variance in well-being. A health-promoting lifestyle predicted greater well-being both directly and indirectly. Among the mediators, depression and sleep quality showed significant indirect effects, whereas anxiety did not play a statistically significant mediating role. Overall, the findings confirm that a health-promoting lifestyle is associated with lower depressive symptomatology and better sleep quality, which in turn enhances subjective well-being.

**Conclusion:**

The findings support an integrative model in which a health-promoting lifestyle explains student well-being, highlighting depression and sleep quality as key pathways of influence. These results broaden the understanding of well-being from a multidimensional perspective and provide actionable evidence for designing institutional policies and intervention strategies that promote healthy lifestyle behaviors and well-being, particularly in Latin American settings.

## 1 Introduction

The transition to university represents a critical developmental period characterized by new academic, social, and emotional demands that can significantly influence students’ psychological well-being (Worsley et al., 2021). During this stage, exposure to stressors such as academic overload, adaptation to unfamiliar environments, and social isolation increases the risk of mental health problems and lower academic performance (Liu et al., 2019; Castillo-Díaz et al., 2022). Global estimates from the World Health Organization (WHO) indicate that approximately one in three university students meets the criteria for at least one common mental disorder, highlighting the substantial burden of psychological distress in this population (Auerbach et al., 2018).

Research shows that mental health problems among adolescents and young adults remain highly prevalent, yet often underrecognized due to social stigma, cultural differences, and the lack of standardized assessment methods (Auerbach et al., 2018; Black et al., 2025). Consequently, within the university context, student well-being has become a priority that requires not only the prevention of mental disorders but also the promotion of positive development and the strengthening of institutional support systems (Douwes et al., 2023; Chaudhry et al., 2024). As a result, enhancing students’ mental health and well-being has emerged as a global priority in both education and public health (Auerbach et al., 2018).

Well-being is currently understood as a multidimensional construct encompassing emotional, psychological, and social components, extending beyond the mere absence of illness (Diener et al., 2018). From the perspective of positive psychology, well-being reflects human flourishing and the capacity to experience meaningful relationships, a sense of purpose, and optimal functioning (Martela, 2025). The dual-continuum model of mental health posits that psychological distress and well-being are related yet distinct dimensions, indicating that individuals may exhibit low levels of psychopathological symptoms while simultaneously reporting some degree of well-being (Westerhof and Keyes, 2010; Keyes, 2014). Accordingly, assessing both dimensions concurrently provides a more complete picture of students’ adjustment and psychological functioning. In university contexts, recent studies suggest that students conceptualize well-being as a positive and holistic construct that integrates multiple, interrelated dimensions of their academic, personal, and social lives (Walker, 2022; Douwes et al., 2023).

Among the behavioral and cognitive correlates that consistently contribute to well-being, health-promoting lifestyles have attracted increasing empirical attention as modifiable factors capable of enhancing both physical and psychological outcomes (Ridner et al., 2016). A health-promoting lifestyle is defined as a multidimensional pattern of behaviors that includes physical activity, balanced nutrition, stress management, health responsibility, and interpersonal support (Walker et al., 1987; Pender et al., 1988). Among university populations, the adoption of health-promoting behaviors has been associated with higher levels of psychological well-being, self-efficacy, and resilience (Amiri et al., 2019).

Recent evidence using network analysis further highlights that health-promoting lifestyles are closely interconnected with anxiety, depression, and sleep problems, underscoring their role in significantly enhancing mental health (Sun et al., 2024). Nevertheless, the underlying mechanisms linking health-promoting behaviors to subjective well-being in university students have not yet been fully elucidated. In particular, the potential joint mediation of mental health—encompassing symptoms of depression and anxiety—and sleep quality has been scarcely examined among university students, despite its theoretical and empirical relevance for understanding student well-being. Therefore, it is essential to examine more precisely how these variables interact to shape subjective well-being.

## 2 Literature review

### 2.1 Health-promoting lifestyle and psychological outcomes

Empirical evidence indicates that students who adopt healthier lifestyles tend to experience higher levels of subjective well-being, even after accounting for sociodemographic differences (Amiri et al., 2019, 2023). These findings suggest that health-promoting behaviors—such as balanced nutrition, regular physical activity, and effective stress management—exert a sustained protective influence on mental health and overall life satisfaction. Thus, a health-promoting lifestyle emerges as a critical correlate of well-being among university students, reinforcing its role as a foundational component of adaptive functioning and positive development in academic contexts (Lee and Loke, 2005).

On the other hand, research has shown that a health-promoting lifestyle is associated with lower levels of depressive and anxiety symptoms among university populations. A recent study conducted with Honduran students reported that greater adherence to healthy behaviors was significantly associated with lower levels of psychological distress, acting as a protective factor against emotional discomfort (Castillo-Díaz et al., 2024). This finding aligns with evidence from Chinese university students, where health-promoting lifestyles have been found to correlate negatively with depression, even after adjusting for social support and sociodemographic characteristics (Tang et al., 2021). Furthermore, previous research has documented that physical activity—a core component of a health-promoting lifestyle—is associated with significant reductions in anxiety and depressive symptoms, and with higher levels of psychological well-being (Buecker et al., 2021; Herbert, 2022), highlighting both preventive and therapeutic benefits.

Similarly, the regular practice of health-promoting behaviors—such as engaging in physical activity, maintaining a balanced diet, and adopting self-care routines—has been associated with greater stability of circadian rhythms and a more favorable subjective perception of nighttime rest (Zhang et al., 2023). Consistent with this, recent research has found that students who report higher levels of health-promoting behaviors tend to exhibit more favorable sleep patterns and a lower incidence of fatigue or daytime sleepiness (Alothman et al., 2024; Sun et al., 2024).

### 2.2 Mental health and sleep quality as pathways to well-being

Higher levels of anxiety have been found to be associated with lower scores of subjective well-being, indicating an inverse relationship between the two variables (Liu et al., 2009; Zhu et al., 2023; Benítez-Agudelo et al., 2025). Similarly, studies conducted among university populations have reported that the presence of depressive symptoms is linked to significantly lower levels of subjective well-being (Liu et al., 2009; Rossi et al., 2019). Additional findings from European student samples confirm that mental well-being decreases in the presence of elevated indicators of emotional distress, further reinforcing this pattern (Navarra-Ventura et al., 2024).

On the other hand, sleep quality has been positively associated with subjective well-being, with evidence showing that better perceived sleep quality corresponds to higher levels of well-being (Su and He, 2023). Intraindividual temporal analyses among university students have revealed concurrent associations between daily variations in sleep and well-being (McGowan et al., 2023). Moreover, findings indicate that from the very first semester of university, shorter sleep duration and greater variability in sleep patterns are linked to poorer indicators of well-being (Bono and Hill, 2022). Taken together, these findings support the role of mental health and sleep quality as key pathways through which psychological functioning and daily experiences shape students’ well-being. However, the complexity of these interrelations remains only partially understood, pointing to the need for more integrated and context-sensitive analyses.

### 2.3 Knowledge gap and study rationale

Despite the arguments presented above, the interplay among health-promoting behaviors, mental health, sleep quality, and well-being in university populations remains insufficiently understood. Moreover, most research on well-being has been conducted in high-income countries, typically involving populations whose structural, cultural, and economic conditions differ substantially from those of Latin American contexts (Thomas et al., 2021; Sollis et al., 2024; Roy et al., 2025). Considering these factors, it is essential to generate context-specific evidence on the factors that may contribute to student well-being.

In response to this gap, the present study aims to evaluate a structural equation model (SEM) in which a health-promoting lifestyle is specified as a predictor of well-being, with affective symptomatology (depression and anxiety) and sleep quality included as mediating variables among Honduran university students. Building on the empirical evidence and conceptual framework outlined, this model examines both direct and indirect pathways to well-being, addressing the limitations of prior research that has often considered these variables in isolation. The SEM approach enables the combine estimation of direct and indirect effects among observed and latent variables (Beran and Violato, 2010), offering a more comprehensive understanding of the behavioral, psychological, and physiological mechanisms underlying well-being.

**Figure 1:**
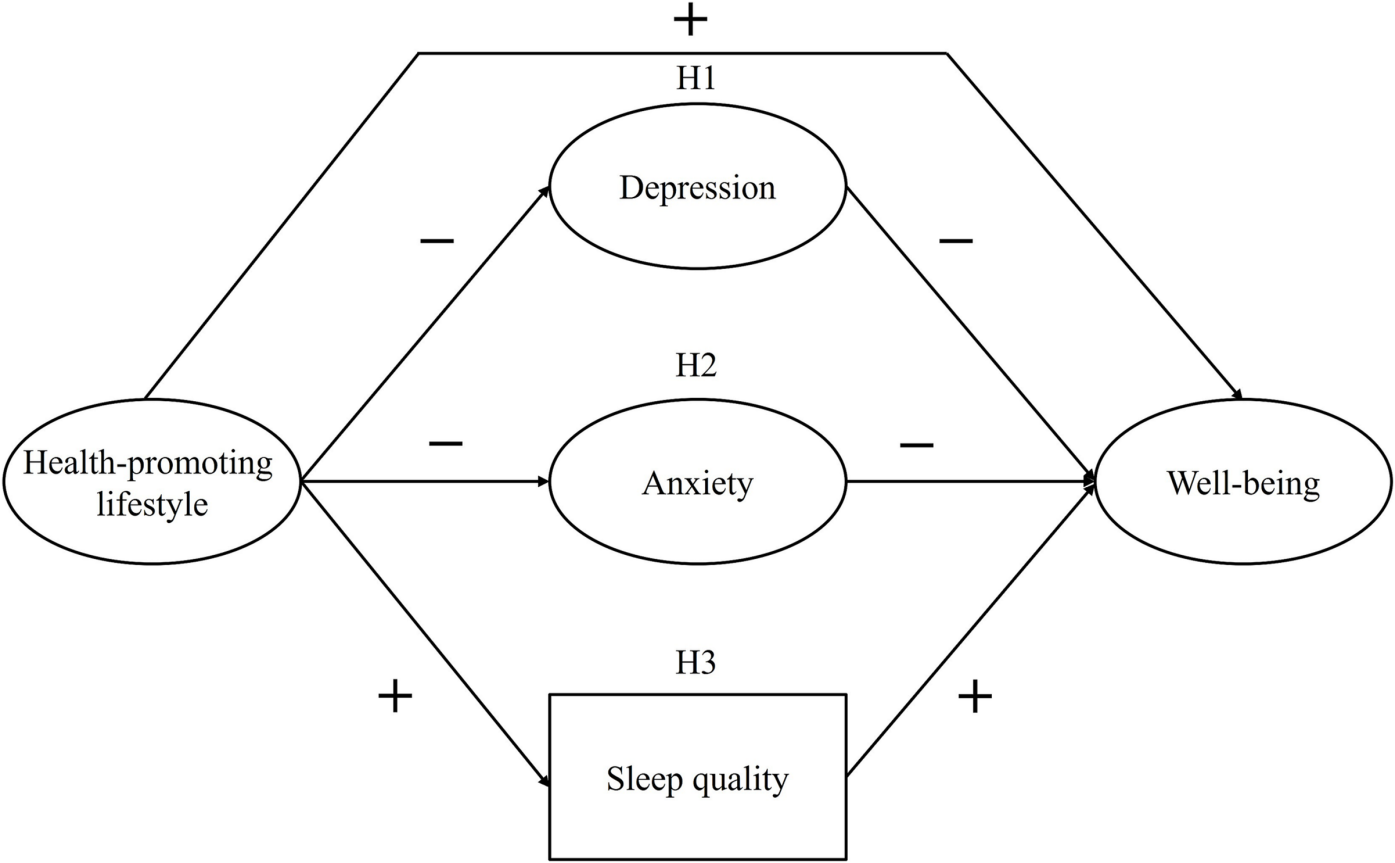
Hypothesized structural model of well-being. H1 = depression as mediator; H2 = anxiety as mediator; H3 = sleep quality as mediator.

Figure 1 depicts the hypothesized structural model. Based on prior evidence, health-promoting lifestyle is expected to positively predict well-being and sleep quality and negatively predict anxiety and depressive symptoms. In turn, anxiety and depressive symptoms are expected to negatively predict well-being, whereas sleep quality is expected to positively predict it. With regard to the mediational pathways, the following hypotheses are proposed:

*H1*: Depressive symptoms mediate the relationship between health-promoting lifestyle and well-being.

*H2*: Anxiety symptoms mediate the relationship between health-promoting lifestyle and well-being.

*H3*: Sleep quality mediates the relationship between health-promoting lifestyle and well-being.

The proposed approach is particularly relevant in contemporary psychological and health research, enabling the examination of complex relationships among variables by integrating behavioral, cognitive, and emotional dimensions. The findings may serve as valuable input for identifying patterns of association that can inform psychoeducational interventions and institutional policies aimed at promoting comprehensive health and enhancing the well-being of higher education students.

## 3 Methods

### 3.1 Design and setting

This study employed a non-experimental, analytical cross-sectional design. It was conducted in a higher education context at a Honduran public macro-university with nationwide coverage.

### 3.2 Participants

The population of the study was composed of Honduran university students from the 2024 and 2025 first-year cohorts, which together comprised 22,850 students (13,379 in 2024 and 9,471 in the first academic term of 2025). Through a non-probabilistic sampling strategy, a total of 6,825 students participated voluntarily; however, 121 were excluded for not meeting the inclusion criteria, which required being a first-year student, aged 18 years or older, enrolled in any academic discipline offered by the university across all campuses nationwide, and providing attentive and non-random responses. Further details on data quality control are described in the Procedures section. The final sample in this study consisted of 6,704 university students, representing 29.3% of the total first-year student population. Sociodemographic characteristics of the sample are summarized in Table 1.

**Table 1:** Sociodemographic characteristics of the sample.

Variables	*n*	%
Gender		
Female	4,367	65.14
Male	2,307	34.41
Another gender identity	30	0.45
University site		
Main Campus (Tegucigalpa)	4,411	65.80
Primary regional campus (San Pedro Sula)	1,117	16.66
Medium and small regional centers	1,176	17.54
Field of study		
Social sciences, humanities, and arts	1,377	20.54
Engineering and physical–mathematical sciences	1,016	15.15
Economics and administrative sciences	3,107	46.35
Biological and health sciences	1,204	17.96
Age (years)	*M* = 20.59	*SD* = 3.48

*M*, Mean; *SD*, Standard deviation.

### 3.3 Measuring instruments

#### 3.3.1 Health-promoting lifestyle

The Health-Promoting Lifestyle Profile II (HPLP-II) was used to assess the adoption of health-promoting behaviors (Walker et al., 1987; Walker and Hill-Polerecky, 1996). Previous research has provided evidence of validity for the full 52-item version among Honduran university students (Castillo-Díaz and Periañez, 2025). However, the present study employed a short version of the scale proposed by Teng et al. (2010). This reduced version consists of 30 items grouped into five dimensions: physical activity (6 items), spiritual growth (6 items), health management (9 items), nutrition (5 items), and health responsibility (4 items). Each item is rated on a four-point Likert scale ranging from 1 (*never*) to 4 (*routinely*), with higher scores on each dimension indicating a greater adoption of health-promoting behaviors. Supplementary Table S1 presents generalized descriptions of the 30 items used in the short-form HPLP-II in both English and Spanish. These summaries provide an overview of the instrument’s content while respecting copyright restrictions. To examine the measurement structure of the HPLP-II short version, we tested a hierarchical model comprising five first-order specific factors and one general second-order factor. The model demonstrated good fit to the data (*χ*^2^ = 10,257.765; df = 400; *p* < 0.001; CFI = 0.977; TLI = 0.975; SRMR = 0.055; RMSEA = 0.061 [90% CI = 0.060, 0.062]). Standardized factor loadings for the first-order factors ranged from *λ* = 0.714 to 0.852 for spiritual growth, λ = 0.733 to 0.787 for physical activity, λ = 0.441 to 0.708 for health management, λ = 0.555 to 0.670 for nutrition, and λ = 0.505 to 0.866 for health responsibility. The general factor showed loadings ranging from λ = 0.663 to 0.865 on the specific factors. Regarding reliability, McDonald’s omega (*Ω*) coefficients for the first-order factors ranged from 0.710 to 0.874, and composite reliability (CR) indices ranged from 0.758 to 0.904. The general factor presented a hierarchical omega (Ωₕₒ) of 0.862, indicating satisfactory reliability at the global level.

#### 3.3.2 Anxiety

The Generalized Anxiety Disorder 7-item Scale (GAD-7) was used to assess symptoms of anxiety (Spitzer et al., 2006). Previous research has provided validity evidence for this instrument among Honduran university students (Landa-Blanco et al., 2025). The scale comprises seven self-report items rated on a four-point Likert scale ranging from 0 (*not at all*) to 3 (*nearly every day*), assessing the frequency of anxiety symptoms during the past 2 weeks. Higher scores reflect greater severity of anxiety symptoms. In this study, a unidimensional measurement model showed good fit to the data (*χ*^2^ = 245.492; df = 14; *p* < 0.001; CFI = 0.998; TLI = 0.998; SRMR = 0.030; RMSEA = 0.050 [90% CI = 0.044, 0.055]), with standardized factor loadings ranging from *λ* = 0.693 to 0.913. The scale demonstrated high internal consistency (*ω* = 0.901; CR = 0.937).

#### 3.3.3 Depression

The Patient Health Questionnaire-9 (PHQ-9) was used to assess depressive symptoms (Kroenke and Spitzer, 2002). The PHQ-9 has been validated among Honduran university students (Landa-Blanco et al., 2025). The scale consists of nine self-report items assessing the frequency of depressive symptoms over the past two weeks. Items are rated on a four-point Likert scale ranging from 0 (*not at all*) to 3 (*nearly every day*), with higher total scores indicating greater severity of depressive symptoms. In this study, a unidimensional measurement model showed good fit to the data (*χ*^2^ = 564.423; df = 27; *p* < 0.001; CFI = 0.996; TLI = 0.995; SRMR = 0.038; RMSEA = 0.054 [90% CI = 0.051, 0.058]), with standardized factor loadings ranging from λ = 0.770 to 0.870. The scale showed high internal consistency (ω = 0.907; CR = 0.946).

#### 3.3.4 Sleep quality

The Single-Item Sleep Quality Scale (SQS) was used to assess perceived sleep quality (Snyder et al., 2018). The SQS is a self-report instrument consisting of a single item rated on a visual analogue scale (VAS) ranging from 0 to 10, with higher scores indicating better perceived sleep quality. Previous studies have provided evidence of validity based on relations with other variables, particularly with other sleep-related measures such as the Pittsburgh Sleep Quality Index (PSQI) and the Morning Questionnaire–Insomnia (MQI) (Snyder et al., 2018; Dereli and Kahraman, 2021), as well as with mental health indicators among Honduran university students (Landa-Blanco et al., 2024). Further empirical support comes from diagnostic accuracy analyses based on ROC curves, which have identified a cut-off score of ≤ 6 with good sensitivity (87.5%) and specificity (74.1%) for detecting poor sleep quality (Badahdah et al., 2025). Regarding temporal stability, test–retest reliability has been reported, with intraclass correlation coefficients (ICC) ranging from 0.62 to 0.82 (Snyder et al., 2018; Dereli and Kahraman, 2021).

#### 3.3.5 Well-being

The World Health Organization Well-Being Index (WHO-5) was used to assess subjective well-being (World Health Organization, 1998; Topp et al., 2015). The scale consists of five self-report items that measure the frequency of positive feelings experienced during the past 2 weeks. Each item is rated on a six-point Likert scale ranging from 0 (*at no time*) to 5 (*all of the time*), with higher scores indicating greater well-being. In this study, a unidimensional measurement model showed good fit to the data (*χ*^2^ = 44.293; df = 4; *p* < 0.001; CFI = 0.999; TLI = 0.999; SRMR = 0.011; RMSEA = 0.039 [90% CI = 0.029, 0.049]), with standardized factor loadings ranging from *λ* = 0.778 to 0.882. The scale demonstrated high internal consistency (*ω* = 0.884; CR = 0.913).

### 3.4 Procedures

Data collection was conducted between January 2024 and June 2025 through online self-administered questionnaires distributed via institutional communication channels, academic events, and classroom spaces regularly attended by first-year university students. The dataset used in this study forms part of a larger research project entitled “*Psychosocial determinants of well-being and quality of life in university students: a diagnostic and longitudinal evaluation*.” All study procedures strictly adhered to the principles of the Declaration of Helsinki and local legislation. The larger research project received ethical approval from the Ethics Committee of the Faculty of Social Sciences at the National Autonomous University of Honduras (Ref. CEIFCS-2024-P01). Participation was entirely voluntary, and digital informed consent was required prior to enrollment.

To ensure the methodological integrity and quality of the data, several quality control procedures were implemented. The online form included embedded attention check items (e.g., “Select option 4 for this question”) and response pattern screening to identify and exclude cases exhibiting straightlining or repetitive response behaviors across multiple scales (e.g., consistently selecting the same option throughout the questionnaire). In addition, minimum completion time thresholds were applied to detect inattentive or automated responses. Specifically, responses completed in less than 15 min—equivalent to approximately 30% of the mean completion time of 49 min observed during the pilot and initial data collection—were flagged for removal. The combined use of attention checks, response pattern detection, and response time screening follows best practices for identifying insufficient effort responding and improving data validity in online questionnaire research (Brühlmann et al., 2020).

### 3.5 Data analysis

As a preliminary step, and considering that all study variables were collected through self-report measures at a single time point, the potential presence of common method bias was examined. To this end, Harman’s single-factor test was conducted by means of an exploratory factor analysis (EFA) including all items from the instruments employed in this study. Following conventional criteria, if the first factor accounted for less than 40% of the total variance, the presence of a severe common method bias was deemed unlikely (Podsakoff et al., 2003).

Descriptive statistics for all variables were computed, including means, standard deviations, minimum and maximum values, histograms, skewness, and kurtosis. Subsequently, bivariate correlations among the main study variables were estimated using the factor scores derived from the measurement models of each instrument (HPLP-II, PHQ-9, GAD-7, and WHO-5), along with the observed score from the SQS. In the case of the HPLP-II, only the correlation for the general factor was reported, as the hierarchical model conceptualizes first-order factors as indicators of the overarching construct.

To test the study hypotheses, a structural equation modeling (SEM) approach was employed. SEM simultaneously integrates measurement models—where latent variables account for their respective observed indicators—and a structural model that estimates the direct and indirect relationships among latent constructs. The measurement models for each latent variable (health-promoting lifestyle, anxiety, depression, and well-being) were assessed through item-level confirmatory factor analyses (CFA), with detailed results reported in the instrument descriptions. Given that sleep quality was represented by a single item, it was analyzed using its observed scores. The conventional sequence of steps for conducting structural equation modeling—specification, identification, estimation, evaluation, and modification—was implemented in accordance with established methodological literature (Kline, 2023).

Figure 1 depicts the tested structural model. The specification of the model indicates a multiple mediation structure in which health-promoting lifestyle predicts well-being both directly and indirectly through depressive symptoms, anxiety symptoms, and sleep quality. The weighted least squares mean- and variance-adjusted (WLSMV) estimator was used for model estimation (Schumacker and Lomax, 2015). Model fit was evaluated using the following indices: comparative fit index (CFI) and Tucker–Lewis index (TLI) values ≥ 0.90 were considered acceptable and ≥ 0.95 indicative of good fit; standardized root mean square residual (SRMR) and root mean square error of approximation (RMSEA) values ≤ 0.08 were regarded as acceptable and ≤ 0.06 as evidence of good fit (Hu and Bentler, 1999; Marsh et al., 2004). We examined direct effects on each endogenous variable, specific indirect effects (via each mediator), total indirect effects (summed across mediators), and overall effects (direct plus indirect). In addition, the proportion of the total effect mediated by each path was computed to facilitate interpretation. Given the large sample size, the delta method was deemed appropriate for computing 95% confidence intervals for all structural effects (Sim et al., 2022).

All analyses were conducted in R (version 4.4.2) using the RStudio environment (version 2025.5.1.513). The *skimr* package (version 2.1.5) (Waring et al., 2022) was used for descriptive analyses, whereas *semTools* (version 0.5–6) (Jorgensen et al., 2022) and *lavaan* (version 0.6–16) (Rosseel, 2012) were employed for the estimation of measurement and structural models.

## 4 Results

### 4.1 Common method bias

As part of the preliminary analyses, common method bias was examined. The EFA results indicated that eight factors had eigenvalues greater than 1, and the variance explained by the first factor was 28.49%, which is below the critical threshold of 40%. These findings suggest that no severe common method bias was present in the data.

### 4.2 Descriptive statistics of the variables

Table 2 presents the descriptive statistics for the variables included in the study. Skewness and kurtosis values fell within the acceptable range (−2 to 2), indicating no severe deviations from normality (Mishra et al., 2019). Regarding health-promoting lifestyle, scores on the HPLP-II exhibited a tendency toward moderate values for both the total score and the subscales of health management and nutrition. The physical activity subscale displayed scores close to the upper limit, whereas the spiritual growth and health responsibility dimensions showed distributions tending toward moderate-to-lower scores on their respective scales. On the other hand, anxiety and depression presented a clear tendency toward lower scores, while sleep quality and well-being displayed distributions with moderate-to-high values.

**Table 2:** Descriptive statistics of the variables.

Variables	Mean	SD	Min.	Max.	Histogram	Skewness	Kurtosis
Health-promoting lifestyle	70.858	14.644	30	120	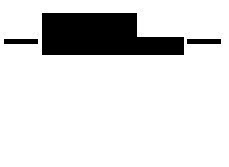	0.302	0.105
Physical activity	17.527	3.899	6	24	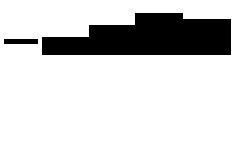	−0.297	−0.455
Spiritual growth	12.331	4.481	6	24	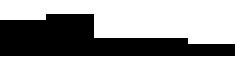	0.601	−0.246
Health management	21.369	4.955	9	36	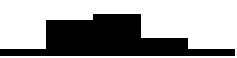	0.285	−0.075
Nutrition	12.063	2.941	5	20	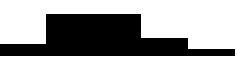	0.309	−0.058
Health responsibility	7.568	2.444	4	16	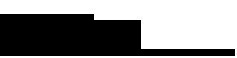	0.664	0.350
Anxiety	4.586	5.355	0	21	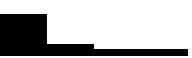	1.276	0.864
Depression	5.330	6.346	0	27	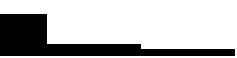	1.339	1.119
Sleep quality	6.823	1.986	0	10	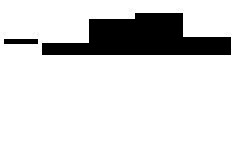	−0.467	0.137
Well-being	15.636	5.546	0	25	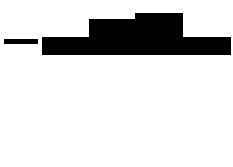	−0.371	−0.527

### 4.3 Bivariate correlations among main variables

Table 3 presents the bivariate correlations among the five main variables of the study. Well-being showed positive correlations with health-promoting lifestyle (*r* = 0.377, *p* < 0.001) and sleep quality (*r* = 0.408, *p* < 0.001), whereas it was negatively correlated with anxiety (*r* = −0.416, *p* < 0.001) and depression (*r* = −0.503, *p* < 0.001). In turn, health-promoting lifestyle was positively associated with sleep quality (*r* = 0.231, *p* < 0.001) and negatively associated with anxiety (*r* = −0.152, *p* < 0.001) and depression (*r* = −0.239, *p* < 0.001). Finally, anxiety and depression were strongly and positively correlated with each other (*r* = 0.744, *p* < 0.001), and both were negatively associated with sleep quality (anxiety: *r* = −0.323, *p* < 0.001; depression: *r* = −0.403, *p* < 0.001).

**Table 3:** Correlations between main variables.

Variables	1	2	3	4	5
1. Health-promoting lifestyle	1				
2. Anxiety	−0.152***	1			
3. Depression	−0.239***	0.744***	1		
4. Sleep quality	0.231***	−0.323***	−0.403***	1	
5. Well-being	0.377***	−0.416***	−0.503***	0.408***	1

***p < 0.001.

### 4.4 Structural equation modeling

Table 4 presents the results of the hypothesized and final structural models. The initial tested model showed an unacceptable fit to the data, with TLI values below 0.90 and SRMR and RMSEA values exceeding 0.08. To optimize the overall model fit, modification indices (MIs) were examined, revealing a theoretically justified residual covariance between anxiety (GAD-7) and depression (PHQ-9). Incorporating this covariance substantially improved model fit (MI = 86,186.00; expected parameter change [EPC] = −1.76). After this re-specification, the final model showed an acceptable and substantially improved fit to the data (*χ*^2^ (1262) = 35,007.519, *p* < 0.001; CFI = 0.972; TLI = 0.971; SRMR = 0.065; RMSEA = 0.063, 90% CI [0.063, 0.064]).

**Table 4:** Fit indices of the predictive model of well-being.

Model	*χ*^2^ (df)	CFI	TLI	SRMR	RMSEA (90% CI)
Hypothesized model	123069.262 (1263)***	0.900	0.895	0.127	0.120 (0.119, 0.121)
Final model	35007.519 (1262)***	0.972	0.971	0.065	0.063 (0.063, 0.064)

*χ*^2^, chi-square test; df, degrees of freedom; CFI, Comparative fit index; TLI, Tucker–Lewis index; SRMR, Standardized root mean square residual; RMSEA, Root mean square error of approximation; CI, Confidence interval; ***p < 0.001.

**Figure 2:**
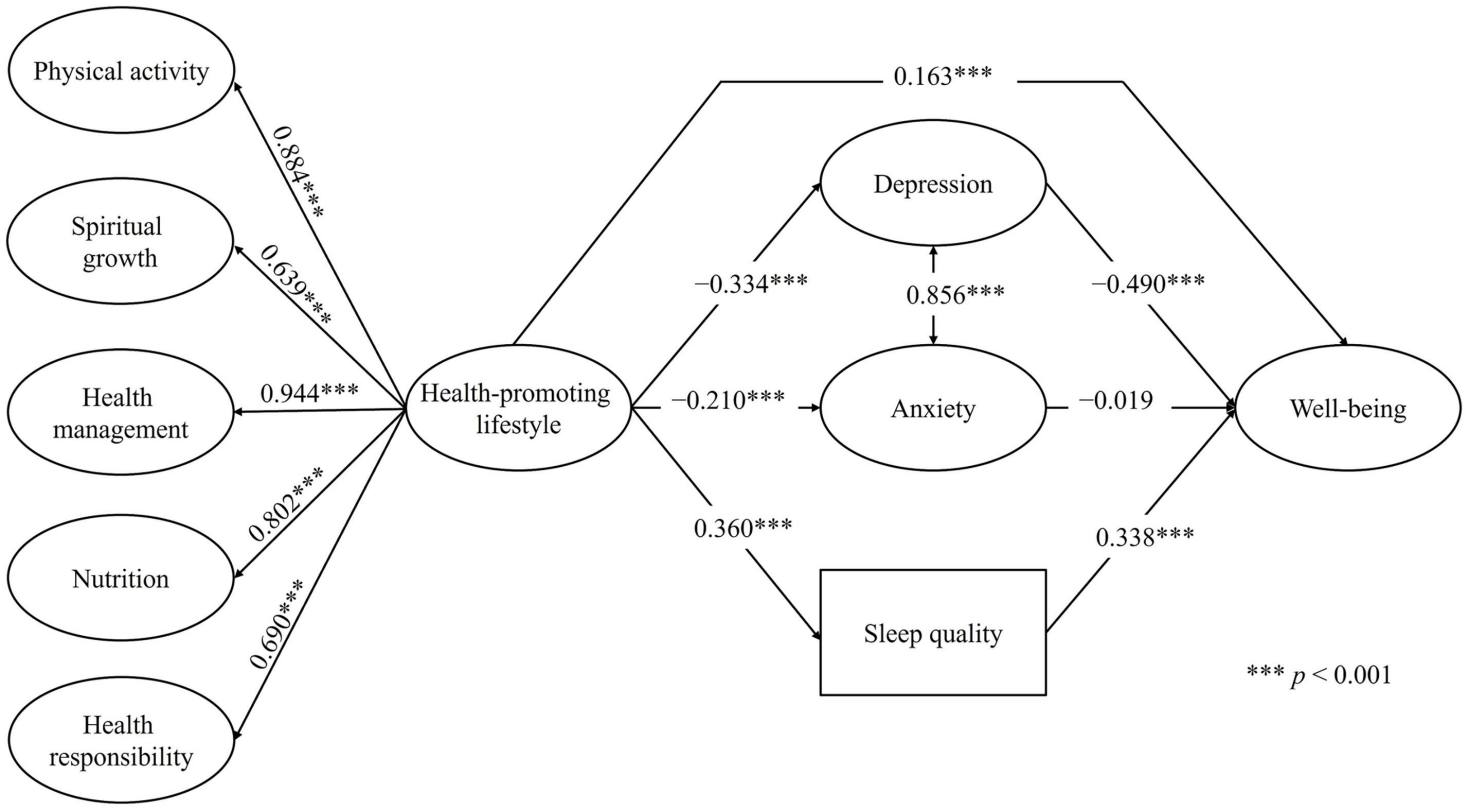
Final structural equation model with direct effects among the study variables. For the sake of clarity and space, the observed indicators of the latent variables are not displayed.

Figure 2 presents the final structural model, showing the standardized coefficients of the direct effects among the constructs. Within the SEM framework, the second-order latent factor of health-promoting lifestyle showed strong and statistically significant loadings on its first-order factors, ranging from 0.639 to 0.944 (*p* < 0.001). The results indicated that health-promoting lifestyle negatively predicted anxiety (*β* = −0.210, [95% CI = −0.236, −0.183], *p* < 0.001) and depression (*β* = −0.334, [95% CI = −0.359, −0.309], *p* < 0.001), and positively predicted sleep quality (*β* = 0.360, [95% CI = 0.337, 0.383], *p* < 0.001) and well-being (*β* = 0.163, [95% CI = 0.134, 0.192], *p* < 0.001). In turn, depression negatively predicted well-being (*β* = −0.490, [95% CI = −0.574, −0.432], *p* < 0.001), whereas sleep quality positively predicted well-being (β = 0.338, [95% CI = 0.316, 0.359], *p* < 0.001). The association between anxiety and depression was positive and strong (*β* = 0.856, [95% CI = 0.845, 0.866], *p* < 0.001). However, the model showed that the direct effects of anxiety on well-being were not statistically significant (*β* = −0.019, [95% CI = −0.075, 0.036], *p* = 0.489).

Table 5 presents the standardized specific indirect, path-specific combined, and total effects. Regarding the specific indirect effects, health-promoting lifestyle significantly predicted well-being through depression (*β* = 0.164, [95% CI = 0.141, 0.186], *p* < 0.001) and through sleep quality (*β* = 0.122, [95% CI = 0.111, 0.132], *p* < 0.001), whereas the pathway through anxiety was not significant (*β* = 0.004, [95% CI = −0.008, 0.016], *p* = 0.491). The total indirect effect, considering all mediators jointly, was significant (*β* = 0.289, [95% CI = 0.269, 0.310], *p* < 0.001).

**Table 5:** Standardized specific indirect, path-specific combined, and total effects from the final model.

Effect type	*β*	95% CI	*p*
Specific indirect effects			
Health-promoting lifestyle → Depression → Well-being	0.164	0.141, 0.186	< 0.001
Health-promoting lifestyle → Anxiety → Well-being	0.004	−0.008, 0.016	0.491
Health-promoting lifestyle → Sleep quality → Well-being	0.122	0.111, 0.132	< 0.001
Total indirect effect (sum of all mediators)	0.289	0.269, 0.310	< 0.001
Path-specific combined effects (direct + indirect via each mediator)			
Health-promoting lifestyle (direct + via depression) → Well-being	0.326	0.298, 0.354	< 0.001
Health-promoting lifestyle (direct + via anxiety) → Well-being	0.167	0.133, 0.200	< 0.001
Health-promoting lifestyle (direct + via sleep quality) → Well-being	0.284	0.259, 0.309	< 0.001
Overall effect (direct + all in directs)	0.452	0.429, 0.475	< 0.001

The path-specific combined effects on well-being (direct + indirect via each mediator) indicated values of *β* = 0.326 (95% CI = 0.298, 0.354; *p* < 0.001) for the depression pathway, *β* = 0.167 (95% CI = 0.133, 0.200; *p* < 0.001) for the anxiety pathway, and *β* = 0.284 (95% CI = 0.259, 0.309; *p* < 0.001) for the sleep quality pathway. The total effect (direct + all indirect effects) of health-promoting lifestyle on well-being was *β* = 0.452 (95% CI = 0.429, 0.475; *p* < 0.001). Regarding the mediated proportion of the total effect, 36.2% of the overall effect was explained through depression (*p* < 0.001), 26.9% through sleep quality (*p* < 0.001), and only 0.9% through anxiety, the latter being non-significant (*p* = 0.490). Finally, the coefficients of determination (*R*^2^) indicated that the model accounted for 61.6% of the variance in well-being, 13.0% in sleep quality, 4.4% in anxiety, and 11.2% in depression.

## 5 Discussion

This study aimed to examine the empirical plausibility of a structural equation model specifying a health-promoting lifestyle as a predictor of subjective well-being, with mental health indicators (depressive and anxiety symptoms) and sleep quality serving as mediating variables. The results showed that the final structural model demonstrated good fit to the data. The findings from the final model integrating health-promoting lifestyle, anxiety, depression, and sleep quality substantially explained university students’ subjective well-being (*R*^2^ = 61.6%). This evidence broadens the understanding of well-being beyond partial approaches focused solely on mental health or behavioral habits. This result aligns with contemporary multidimensional conceptions of well-being (Diener et al., 2018), the notion of human flourishing (Martela, 2025), and the dual-continua model, which distinguishes between the absence of psychopathology and the presence of positive functioning (Westerhof and Keyes, 2010; Keyes, 2014).

Regarding the direct effects of the model, a health-promoting lifestyle showed a positive direct effect on well-being (*β* = 0.163, *p* < 0.001), reinforcing its value as a modifiable and protective factor in university life. This result is consistent with previous evidence showing that students who adopt healthy behaviors report higher levels of well-being and life satisfaction (Lee and Loke, 2005; Amiri et al., 2019). Conversely, a health-promoting lifestyle was negatively associated with depressive (*β* = −0.334, *p* < 0.001) and anxiety symptoms (*β* = −0.210, *p* < 0.001), and positively associated with sleep quality (*β* = 0.360, *p* < 0.001), reflecting a more favorable emotional and physiological profile. These findings are consistent with prior studies demonstrating that a health-promoting lifestyle functions as a protective factor against psychological distress (Tang et al., 2021; Castillo-Díaz et al., 2024), that physical activity significantly reduces symptoms of anxiety and depression (Buecker et al., 2021), and that healthy routines promote circadian rhythm stability and better sleep perception (Zhang et al., 2023).

The hypothesis 1 was supported, as depression significantly mediated the relationship between health-promoting lifestyle and well-being (*β* = 0.164, *p* < 0.001), confirming it as the key psychological pathway in this model. This mediation accounted for 36.2% of the total effect and aligns with studies indicating that depressive symptomatology is associated with lower well-being among university students (Rossi et al., 2019). From the perspective of the dual-continua model (Westerhof and Keyes, 2010), the results suggest that enhancing health-promoting behaviors may reduce depressive symptoms and, consequently, foster positive functioning. The identification of depression as a significant mediator highlights its importance as a key mental health target for the implementation of preventive and psychoeducational strategies.

Concerning hypothesis 2, anxiety did not show a significant mediating effect in the relationship between health-promoting lifestyle and well-being. The direct effect (*β* = −0.019) and the indirect effect (*β* = 0.004) were not statistically significant (*p* > 0.05), suggesting that, unlike depression, anxiety does not operate as a mediator of well-being in this population. Although previous literature has documented an inverse association between anxiety and well-being (Liu et al., 2009), the findings of the present study indicate that depression exerts a more stable influence on well-being. One plausible explanation for this pattern is the high covariance between depression and anxiety (*β* = 0.856, *p* < 0.001). This strong overlap may have absorbed shared variance related to emotional distress, thereby reducing the unique explanatory power of anxiety as an independent mediator.

The absence of a significant mediating role of anxiety is also consistent with a study conducted among Brazilian and Argentine university students, which found that positive affect—a core dimension of well-being—was not significantly associated with state anxiety (Dias Lopes et al., 2020). This pattern suggests that the emotional dimensions underlying anxiety may be less closely linked to perceived well-being in certain Latin American contexts, possibly reflecting cultural differences in how anxiety is experienced and expressed. Nevertheless, such interpretations warrant further cross-cultural and longitudinal examination. Another factor that may contribute to the nonsignificant mediation is the nature of the assessment of anxiety. The present study employed a measure of general anxiety symptoms (GAD-7) that does not differentiate between state and trait components. These distinctions may capture different theoretical mechanisms and could yield different mediation patterns. Future research should therefore examine whether the mediational role of anxiety varies depending on its conceptualization and measurement.

With respect to hypothesis 3, in which sleep quality was expected to mediate the relationship between health-promoting lifestyle and well-being, this pathway was supported (*β* = 0.122, *p* < 0.001). This finding reinforces the plausibility of a key physiological pathway, aligning with evidence linking better sleep quality to higher levels of well-being among university students (Bono and Hill, 2022; Su and He, 2023), as well as with studies showing that healthy behaviors contribute to circadian rhythm stability and reduced fatigue (Zhang et al., 2023; Alothman et al., 2024; Sun et al., 2024).

In addition to its theoretical contributions, this study provides contextually grounded evidence based on a large (*n* = 6,704) and diverse sample of Honduran university students. Most studies on well-being originate from industrialized and high-income countries (Sollis et al., 2024). Therefore, the present findings help to balance the geographical bias in the literature and offer culturally relevant and context-sensitive insights from a lower–middle-income country such Honduras. Although the associations modeled here are consistent with prior international research, the finding that depression and sleep quality—but not anxiety—operate as significant pathways offers a distinctive contribution by highlighting patterns that may be particularly relevant in Latin American university contexts.

From an applied perspective, the three statistically significant pathways identified in relation to well-being—namely, the direct effect of health-promoting lifestyle and the indirect effects through depression and sleep quality—highlight specific areas for intervention within higher education institutions. Universities can integrate the promotion of health-promoting behaviors into their well-being policies and curricula, strengthen psychosocial support systems, and enhance early detection strategies for depressive symptoms. Moreover, incorporating preventive and psychoeducational initiatives focused on sleep hygiene may provide accessible and cost-effective avenues for fostering student well-being.

Despite the valuable contributions of this study, several important limitations should be acknowledged, as they may guide future research directions. First, the use of a convenience sampling approach—although it included a large sample of first-year students from a major public university in Honduras—limits the generalizability of the findings. Additionally, the sample was composed predominantly of female students from the central campus and the field of economics. While this distribution reflects the demographic structure of the university population, future studies should employ more sophisticated probabilistic sampling methods and include participants from other higher education institutions and diverse sociocultural backgrounds to enhance external validity and contextual representativeness.

Second, the data in this study were derived from self-report instruments collected at a single time point. Although several procedures were implemented to ensure data quality, including steps to minimize common method bias, future research should employ multimethod strategies for data collection. Such approaches would more robustly mitigate the inherent social desirability bias associated with self-report measures and allow for cross-validation of the findings.

Third, the instruments used in this study are internationally validated tools that demonstrated adequate evidence of validity and reliability within the analyzed measurement models. However, regarding the use of the SQS, it was not possible to examine the structural validity and measurement error of this scale in the study sample. Future research should explore the psychometric properties of the SQS more thoroughly in Latin American populations. Moreover, subsequent studies could benefit from incorporating brief sleep quality measures as alternative tools, enabling the analysis and inclusion of this variable from a latent-variable perspective. In addition, it would be valuable to complement self-report instruments with objective and clinimetric approaches—such as actigraphy, polysomnography, or standardized risk scoring methods—to obtain more accurate and multidimensional indicators of sleep quality within predictive models of well-being.

Fourth, in line with the instruments employed, the short version of the HPLP-II was incorporated into the structural model through its hierarchical factorial structure, consisting of five first-order factors and one second-order general factor. Although this measurement model demonstrated good fit, adequate factor loadings, and high hierarchical omega reliability, the present study did not examine the direct and indirect effects of the individual HPLP-II dimensions on well-being through the mediators. Consequently, it was not possible to determine the specific predictive contribution of each dimension to well-being. Future studies incorporating bifactor measurement models and explanatory analyses of these dimensions could provide more precise predictive insights and, in turn, inform the design of more targeted and effective interventions to promote university students’ well-being.

Fifth, given the cross-sectional design of this study, causal inferences regarding the direct and indirect relationships among the study variables cannot be established. Future research would benefit from employing longitudinal SEM designs that allow for the examination of temporal relationships among the variables and capture intraindividual variations in the analyzed constructs as first-year students progress through their university experience. This is particularly relevant in higher education contexts, where students’ academic trajectories may shape long-term well-being outcomes (du Toit et al., 2022).

Sixth, the SEM model analyzed in this study did not include control variables such as age, gender, socioeconomic status, or other individual and contextual factors that may influence the results. Future research should examine the model while accounting for these variables. Moreover, given the complex and interconnected nature of psychological and behavioral phenomena, the model proposed in this study should be corroborated in subsequent research that verifies the findings and the mediation pathways across cross-cultural contexts.

## 6 Conclusion

The findings of this study provide empirical support for an integrative explanatory model in which health-promoting behaviors predict university students’ subjective well-being both directly and indirectly through mechanisms related to mental health—particularly depressive symptomatology—and sleep quality. The absence of a significant mediating effect of anxiety suggests that depressive symptoms and sleep quality may play a more decisive role than anxious activation in shaping the well-being experience of this population. This pattern may reflect culturally specific tendencies in the experience and expression of anxiety in Latin American settings. Furthermore, drawing on a large university student sample, the final model accounted for over 60% of the variance in well-being, underscoring the model’s explanatory power and empirical relevance.

From a theoretical standpoint, this study expands our understanding of the determinants of well-being in university settings by integrating health behaviors, emotional indicators, and physiological factors into a single predictive model. The evidence supports the relevance of addressing well-being from a multifactorial perspective that recognizes the interdependence between healthy lifestyle behaviors, affective symptoms, and sleep quality. Furthermore, identifying depression and sleep quality as statistically significant mediators provides elements for refining conceptual models of student well-being, differentiating the predominant emotional mechanisms in university populations from specific socioeconomic contexts.

Finally, as part of the practical implications of the findings, the results provide valuable input for the design of preventive strategies and psychoeducational interventions in higher education. Promoting healthy lifestyle behaviors, strengthening institutional efforts for the early detection and management of depressive symptoms, and fostering healthy sleep habits may jointly contribute to enhancing students’ well-being. Moreover, since this evidence was generated in a lower–middle-income country, the study provides a perspective that is underrepresented in the international literature, offering contextualized information that can inform more equitable and culturally sensitive university health and well-being policies.

## Data Availability

The raw data supporting the conclusions of this article will be made available by the authors, without undue reservation.
