# Supplementary material for: Health-promoting lifestyle as a predictor of well-being in Honduran university students: a structural equation modeling approach with mental health and sleep quality as mediators
Source: Front Psychol. 2026 Jan 20;16:1735602. doi: 10.3389/fpsyg.2025.1735602 (PMC12864460; doi:10.3389/fpsyg.2025.1735602)
Supplement: Supplementary file 1 [file Table_1.DOCX]

Supplementary Material

# Supplementary Table

**Table S1**. Generalized item content of the 30-item short-form Health-Promoting Lifestyle Profile II (HPLP-II)

| **Item number**  (Walker et al., 1987) | **Generalized item description** | |
| --- | --- | --- |
|  | **English** | **Spanish** |
| **Physical activity** |  |  |
| 4 | Follow exercise program | *Sigue un programa de ejercicio* |
| 10 | Vigorous exercise 3 times/week | *Realiza ejercicio vigoroso tres veces por semana* |
| 16 | Light to moderate physical activity | *Realiza actividad física ligera o moderada* |
| 22 | Attend leisure time | *Participa en actividades recreativas* |
| 28 | Do stretching exercise | *Realiza ejercicios de estiramiento* |
| 34 | Get exercise daily activities | *Realiza actividades físicas cotidianas* |
| **Spiritual growth** |  |  |
| 6 | Positive growing/changing | *Siente un crecimiento y cambio positivo* |
| 12 | Life has purpose | *Reconoce que la vida tiene un propósito* |
| 18 | Look forward to future | *Mira hacia el futuro con optimismo* |
| 30 | Long-term goals | *Establece metas a largo plazo* |
| 42 | Know what is important | *Identifica lo que es importante en la vida* |
| 52 | New experience and challenge | *Se expone a nuevas experiencias y desafíos* |
| **Health management** |  |  |
| 7 | Respect accomplishment | *Reconoce y valora logros* |
| 11 | Daily relaxation time | *Dedica tiempo diario a relajarse* |
| 23 | Pleasant bedtime thoughts | *Mantiene pensamientos agradables antes de dormir* |
| 25 | Express concern/love | *Expresa preocupación o afecto* |
| 31 | Touch/am touched | *Busca o acepta contacto afectivo* |
| 13 | Maintain meaningful interpersonal relationships | *Mantiene relaciones interpersonales significativas* |
| 41 | Meditation/relaxation | *Practica meditación o relajación* |
| 43 | Get support from network | *Busca apoyo en su red social* |
| 47 | Prevent tiredness | *Previene el cansancio mediante hábitos saludables* |
| **Nutrition** |  |  |
| 20 | Eat fruit | *Consume frutas* |
| 26 | Eat vegetables | *Consume vegetales* |
| 32 | Eat milk, yogurt or cheese | *Consume leche, yogur o queso* |
| 38 | Eat 5 food groups | *Consume alimentos de los cinco grupos* |
| 50 | Eat breakfast | *Desayuna regularmente* |
| **Health responsibility** |  |  |
| 3 | Report symptoms to MD | *Informa síntomas a un profesional de salud* |
| 21 | Question MD/second opinion | *Solicita una segunda opinión médica* |
| 27 | Discuss health concerns | *Discute sobre preocupaciones de salud* |
| 39 | Seek information | *Busca información de salud* |
| *Note.* Item descriptions represent generalized content summaries and do not reproduce the original copyrighted wording of the HPLP-II. The full HPLP-II instrument, originally published by Walker et al. (1987) and Walker & Hill-Polerecky (1996), is available for consultation through the University of Michigan Deep Blue repository, which provides access to the Spanish version of the scale: <https://deepblue.lib.umich.edu/items/2d1c6350-9bf5-4c2e-bda8-424918ce0a92>. The 30-item short-form version used in this study is based on the adaptation proposed by Teng et al. (2010). | | |

# References

Teng, H., Yen, M., and Fetzer, S. (2010). Health promotion lifestyle profile‐II: Chinese version short form. *J Adv Nurs* 66, 1864–1873. doi: 10.1111/j.1365-2648.2010.05353.x

Walker, S. N., and Hill-Polerecky, D. M. (1996). Psychometric evaluation of the Health-Promoting Lifestyle Profile II. *Unpublished manuscript, University of Nebraska Medical Center*.

Walker, S. N., Sechrist, K. R., and Pender, N. J. (1987). The Health-Promoting Lifestyle Profile: development and psychometric characteristics. *Nurs Res* 36, 76–81. doi: 10.1097/00006199-198703000-00002
